# Noninvasive drug adherence monitoring of antipsychotic patients via finger sweat testing

**DOI:** 10.3389/fchem.2023.1245089

**Published:** 2023-08-31

**Authors:** K. Longman, C. Frampas, H. Lewis, C. Costa, R. Nilforooshan, M. Chambers, M. Bailey

**Affiliations:** ^1^ School of Chemistry and Chemical Engineering, University of Surrey, Guildford, United Kingdom; ^2^ Surrey Ion Beam Centre, University of Surrey, Guildford, United Kingdom; ^3^ Abraham Cowley Unit, St Peter’s Hospital, Surrey and Borders Partnership NHS Foundation Trust, Chertsey, United Kingdom; ^4^ Faculty of Health and Medical Sciences, University of Surrey, Guildford, United Kingdom

**Keywords:** finger sweat, noninvasive, antipsychotic, adherence, liquid chromatography mass spectrometry

## Abstract

Collection of finger sweat is explored here as a rapid and convenient way of monitoring patient adherence to antipsychotic drugs. Finger sweat samples (*n* = 426) collected from patients receiving treatment with clozapine, quetiapine and olanzapine were analysed by liquid chromatography mass spectrometry, including a subgroup of patients with paired plasma samples. Finger sweat samples were also analysed from a negative control group and patients who had handled antipsychotic medication only. The finger sweat test (based on the detection of parent drug in one donated sample) was 100% effective in monitoring adherence within commonly prescribed dosing ranges. In comparison to participants who handled the medication only, the test could distinguish between contact and administration through monitoring of the drug metabolite, or the level of parent drug. Additionally, in a subgroup of patients prescribed clozapine, a statistically significant correlation was observed between the mass of parent drug in finger sweat and plasma concentration. The finger sweat technology shows promise as a dignified, noninvasive method to monitor treatment adherence in patients taking antipsychotics.

## 1 Introduction

One of the greatest challenges in the treatment of psychotic disorders is nonadherence to antipsychotic medication, where approximately half of patients do not adhere to their prescribed regime (García et al., 2016). Poor compliance is consistently associated with a high rate of relapse and unfortunate patient outcomes, including increased risk of rehospitalization and suicide (Higashi et al., 2013). Such consequences extend beyond patient welfare, where greater use of emergency services, longer hospital stays and societal effects from violent or criminal behavior incur increased costs to society and healthcare systems.

Atypical antipsychotics such as clozapine (CLZ), quetiapine (QTP) and olanzapine (OLZ) are commonly prescribed for the treatment of schizophrenia and other psychotic and affective disorders. Reportedly more effective than their predecessors, particularly in respect to the negative or depressive symptoms of psychosis, these second-generation medications are less likely to produce extrapyramidal side effects (Gründer et al., 2009). Whilst better tolerated, several adverse effects, most notably metabolic disruption (Pillinger et al., 2020) are associated with these medications, especially at higher dosages (Haddad and Sharma, 2012). Despite the associated risks, antipsychotic medications are considered the primary tool for the alleviation and management of psychotic symptoms.

Various objective (observed administration, electronic pill dispensers and drug measurements) and subjective (self-reporting, clinicians’ opinion) methods have been used to monitor antipsychotic adherence (Velligan et al., 2006; Haddad et al., 2014). Objective methods are considered to provide more accurate measurements than subjective methods. Measurement of antipsychotic drugs and metabolites in biofluids can inform dose adjustment and reduce the risk of adverse effects or toxicity (Skogh et al., 2002; Qi and Liu, 2021), as well as provide information relating to adherence. Traditionally such measurements are performed using serum or plasma (Aravagiri and Marder, 2001; Zhou et al., 2004; Cao et al., 2020; Qi and Liu, 2021). However, analytical methods have been developed for the evaluation of antipsychotics in hair (Weinmann et al., 2002; Günther et al., 2018; 2020), oral (Fisher et al., 2013), and cerebrospinal fluid (Josefsson et al., 2010).

Fingerprints, or finger sweat, offer a noninvasive alternative for drug monitoring. Sample collection is quick, convenient and does not require any specialist materials or training. Storage and transportation of sweat samples is much simpler than more traditional matrices as the samples are not biohazardous. The detection of drugs and their metabolites in fingerprint sweat has been reported previously for both illicit (Jacob et al., 2008; Bailey et al., 2015; Costa et al., 2017; Hudson et al., 2018; Ismail et al., 2018; Czerwinska et al., 2020; Jang et al., 2020) and therapeutic drugs (Goucher et al., 2009; Costa et al., 2021; Ismail et al., 2022). Finger sweat from unwashed hands comprises eccrine sweat, as well as substances handled by a participant. In contrast, finger sweat samples provided after handwashing are understood to be more reflective of eccrine sweat (Jang et al., 2020).

Our previous work (Costa et al., 2021) has shown that QTP was detected in the fingerprints of two patients, but to our knowledge, the presence of other antipsychotic drugs in finger sweat, and the significance of detecting them, has never been reported. Here we seek to explore the prevalence of antipsychotic medication in finger sweat and assess the significance of a ‘*Positive*’ result for the first time.

In this work, we describe a method for the determination of CLZ, QTP and OLZ in finger sweat by liquid chromatography coupled to tandem mass spectrometry (LC-MS/MS). We assess the ability of the method to detect the parent drug and metabolite in finger sweat with respect to administered dose. To test whether a patient can falsify the test, we compare these results to participants who have handled the drug only, along with a negative control group. Finally, to assess the quantitative potential of the finger sweat test, we explore the relationship between CLZ and its metabolite, *N*-desmethylclozapine (NDMC) in finger sweat and paired plasma samples.

## 2 Methods

### 2.1 Sample collection

Favorable ethical opinion was obtained from the National Research Ethics Service (NRES-REC reference 18/NE/0071) for the collection of finger sweat and plasma samples from patients at Surrey and Borders Partnership NHS Trust and Sussex Partnership NHS Foundation Trust. Prior to sampling, all patients provided written informed consent for collection and subsequent analysis for drug level determination. Informed consent for study participation included provision for the collection of basic metadata parameters (including treatment regime and time of last dose–see Supplementary Table S1) alongside biological sampling.

The study primarily focused on collection of finger sweat samples from patients receiving treatment with either CLZ (n = 33 patients; 198 samples), QTP (n = 7 patients; 42 samples) or OLZ (n = 20 patients; 120 samples). An additional sub study was performed where patients (n = 11) receiving CLZ also provided paired (time-matched) plasma for comparison to finger sweat. For patients receiving CLZ and QTP, oral administration was prescribed in the range 25—550 and 100—500 mg/day, respectively. For OLZ, the prescribed treatment regime was a combination of oral administration in the range 2.5—20 mg/day and monthly injection in the range 300—420 mg. Information relating to dose and time of last administration included within this study was self-reported by patients. We have therefore assumed truthful reporting and adherence to prescribed medication.

Additional finger sweat samples were collected from a drug naive negative control group (n = 30) as well as participants whose only association with an assigned antipsychotic drug was through touch. To mimic events which may lead to a false positive result, either accidental or intentional, these “contact only” participants (n = 6) were asked to handle antipsychotic medication. The participants were asked to handle whole tablets with their right hand and to touch crushed tablets with the left hand before donating samples from both hands.

#### 2.1.1 Finger sweat collection

Finger sweat collection devices were prepared by mounting a paper substrate (Whatman 1-Chr-grade) atop a glass microscopy slide.

**FIGURE 1:**
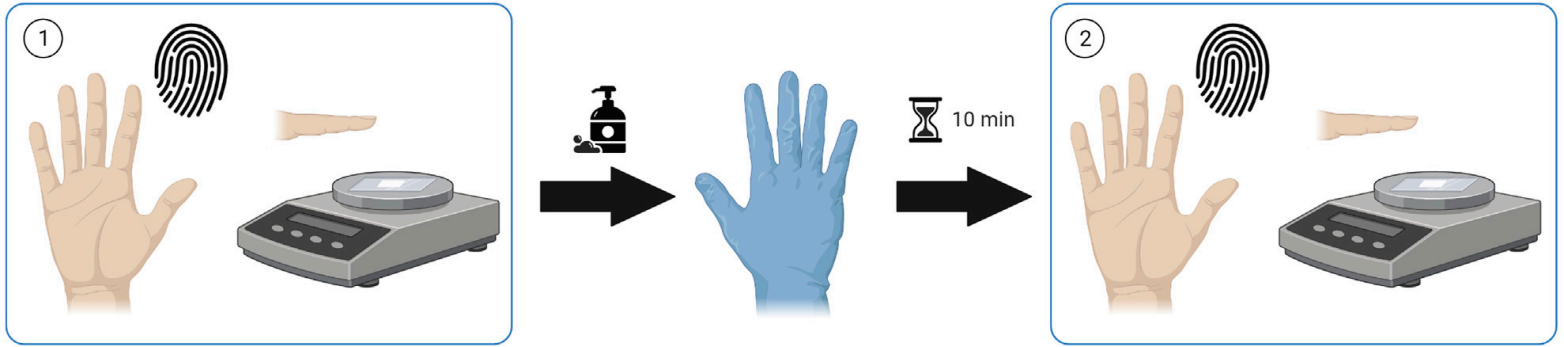
Sample collection procedure. Finger sweat samples collected (1) “as presented” and (2) “after washing”. Created using Biorender.com.

Finger sweat samples were collected from patients receiving antipsychotic medication before and after a hand washing procedure, hereby described as “*as presented*” and “*after washing*” respectively. Unless otherwise specified, finger sweat samples were collected from the index, middle and ring fingers of the right hand. After hand washing with soap and tap water to remove external contamination, patients were required to wear nitrile gloves for a period of 10 min to induce sweating before collection of *after washing* samples (Figure 1).

Finger sweat samples from a drug naive control group (n = 30) were collected from the right index finger only. Collection of samples within drug contact study were collected from both hands after handling of medication. To avoid contact between the two hands, the fingertips were washed separately by a gloved third party.

All samples were collected onto the porous paper substrate using controlled deposition time and pressure, 30 s at a pressure of 800–1,200 g (measured using a generic kitchen scale). Samples were transported at ambient temperature in microscope storage boxes before transfer to—80°C for long-term storage until analysis.

#### 2.1.2 Plasma sample collection

Paired plasma samples were also collected from subgroup of patients (n = 11) receiving treatment with CLZ. Venous blood samples were collected into 3 mL heparinized vials. All samples were centrifuged at 4°C for 10 min (1725 ×*g*) and the resultant plasma was stored at—80 °C until analysis.

### 2.2 Chemicals and reagents

Certified reference materials of antipsychotic drugs (CLZ, QTP, OLZ) their respective metabolites (NDMC, norquetiapine (NQTP), *N*-desmethylolanzapine (DMO)) and deuterated internal standards (IS) (CLZ-d4, QTP-d8, OLZ-d8) were obtained from Sigma Aldrich. Optima™ LC/MS grade acetonitrile (ACN), formic acid (FA), methanol and water were obtained from Fisher Scientific.

### 2.3 Standard curve samples

#### 2.3.1 Finger sweat methodology

A typical standard curve consisted of six standard levels prepared in water and spiked in fixed volume (10 µL) onto chromatography paper (Whatman 1-Chr-grade, 2 × 2 cm) as well as blank papers. Each level was freshly prepared in triplicate before drying at ambient temperature for 1 hour prior to extraction from paper as described in section 2.4.1. Based on proof-of-concept data, standard curve and quality control (QC) samples were prepared using this procedure but at different concentrations depending on analyte. Standards ranged from 60—360 pg for CLZ/NDMC, 30—180 pg for QTP/NQTP and 100—600 pg for OLZ/DMO. Internal standards (IS) were 200 pg, 100 pg and 350 pg, respectively. For assessment of method performance, three QC levels were prepared on paper for each analyte, reflective of lower, middle and upper of the working range. These were 90, 200 and 330 pg for CLZ/NDMC; 40, 100 and 160 pg for QTP/NQTP; and 150, 350 and 550 pg for OLZ/DMO.

For patients with matched plasma samples, the working range was extended to 100—600 pg (IS = 350 pg) for CLZ and NDMC. Three QC levels (150, 350 and 550 pg) were selected to assess the performance of the extended range.

#### 2.3.2 Plasma methodology

For measurement of CLZ and NDMC only, standard curve samples were prepared by spiking pooled plasma in the range of 25—1,500 ng/mL. Three QC levels (75, 500 and 1,200 ng/mL) were prepared in pooled plasma for verification of method performance. An IS solution (100 ng/mL CLZ-d4) was prepared in methanol.

### 2.4 Sample preparation

#### 2.4.1 Finger sweat samples

Prior to extraction, samples prepared on paper (i.e., standard curve or QC samples) or collected onto paper (i.e., finger sweat from patients or drug naïve controls) were spiked with an IS solution (exact mass of IS described in Section 2.3.1) and allowed to dry at ambient temperature for 1 hour. Using sterile tweezers, the papers were transferred into 2 mL centrifuge tubes with 1.5 mL of methanol. The tubes were then centrifuged at 9,500 ×*g* for 2 min. The paper substrate was discarded using sterilized tweezers, and the resultant solvent extract was evaporated to dryness using nitrogen. Samples were reconstituted in 100 µL 50:50 mobile phase A (10 mM ammonium acetate adjusted to pH 4.6 using FA) and mobile phase B (ACN +0.1% FA (v/v)) for analysis.

#### 2.4.2 Plasma samples

All standard, QC and patient plasma samples were prepared using an existing extraction procedure (Qi and Liu, 2021). 50 μL aliquots of plasma were defrosted and prepared by protein precipitation with ACN. To each sample, 150 µL of ACN and 20 µL of IS solution were added and vortexed for 5 min. The mixtures were subjected to refrigerated (4°C) centrifugation at 15,000 rpm for 8 min 40 μL of the resultant supernatant was removed and immersed in 200 µL of water, vortexed for a further minute and transferred into vials for analysis.

### 2.5 Instrumentation and sample analysis

Chromatographic separation was performed using a Thermo Scientific™ Ultimate 3,000 ultra-high-performance liquid chromatography (UHPLC) system equipped with a Kinetex XB-C18 column (100 × 2.1 mm, 5 μm, Phenomenex). The 3-min gradient separation was operated at 30°C with a flow rate of 0.25 mL/min. The starting mobile phase comprised 95% A and 5% B which linearly increased to 80% B at 2 min, held constant for 0.5 min, before returning to the initial composition.

The UHPLC system was coupled to a Thermo Scientific Q Exactive™ Plus Hybrid Quadrupole-Orbitrap™ Mass Spectrometer (MS). The MS was used to scan for all analytes using high resolution full scan (HRFS). MS/MS analysis was performed using data dependent acquisition mode (dd-MS^2^). The operating conditions of both the HRFS and dd-MS^2^ are defined in Supplementary Table S2.

Data acquisition and processing was completed using Thermo Scientific TraceFinder™ software (version 5.0). Statistical analysis was performed using IBM SPSS Statistics software (version 28.0).

### 2.6 Method performance

Method performance was evaluated using the Food and Drug Administration guidelines to determine selectivity, linearity, intra- and inter-day accuracy and precision, limit of detection (LOD) and lower limit of quantification (LLOQ), matrix effect and recovery (finger sweat only), carryover and stability (U.S. Department of Health and Human Services and Food and Drug Administration, 2018). Full details of method performance are provided within the Supplementary Material.

In brief, a linear response was obtained for all drugs and metabolites with R^2^ values of >0.973 when extracted from paper (Supplementary Table S3). Using the initially selected working ranges for qualitative assessment of antipsychotics in finger sweat only, the method was repeatable for analytes extracted on the same day (intra-day relative standard deviation (RSD) < 20%) (Supplementary Table S4). However, for OLZ/DMO, the method showed poor reproducibility when comparing QC samples extracted from paper on non-consecutive days. Therefore, clinical finger sweat samples were run in batches determined by drug type with standard curves extracted on each day of analysis.

Method performance experiments were repeated to assess the performance of the finger sweat method at the higher working range selected for the subset of patients with paired finger sweat and plasma samples. The intra- and inter-day accuracy and precision of CLZ/NDMC extracted from paper and in plasma was assessed using relative error (RE) and RSD. The three QC levels indicated acceptable accuracy and precision (RE and RSD <15%) for both biological matrices (Supplementary Tables S5, S6).

## 3 Results

Chromatograms of drug and metabolite standards are displayed in Supplementary Figure S1. No significant interference with the antipsychotic drugs and metabolites were observed within the paper only blanks. Proof of concept through successful detection and confirmation by dd-MS^2^ was achieved for each of the parent drugs and their metabolites within human samples (see Supplementary Figure S2). All antipsychotics were below the LOD for samples collected from the negative control group (see Supplementary Figure S3).

### 3.1 Detection of antipsychotics in finger sweat

The detection rate of the parent drug and metabolite in all finger sweat samples *versus* per participant is summarized in Table 1. Samples and/or patients are classified as “positive” where the analyte is detected.

**TABLE 1:** Detection of antipsychotic drugs and metabolites in finger sweat only participants, collected “as presented” and “after washing”, defined in all samples and per participant.

Target analyte	Detection in samples, % (n) ^a^		Detection in participants, % (N) ^b^	
	As presented	After washing	As presented	After washing
CLZ ^c^	100 (96)	100 (96)	100 (33)	100 (32)
NDMC ^c^	86 (83)	80 (77)	94 (30)	88 (28)
QTP	100 (21)	100 (21)	100 (7)	100 (7)
NQTP	100 (21)	100 (21)	100 (7)	100 (7)
OLZ	88 (53)	75 (45)	100 (20)	80 (16)
DMO	10 (6)	0 (0)	15 (3)	0 (0)

^a^ “n” defined as the total number of samples per drug.

^b^ “N” defined as the total number of participants per drug where positive detection requires analyte presence in at least one finger.

^c^ Samples from participants prescribed ≥100 mg/day.

#### 3.1.1 Clozapine

CLZ performed most reliably of the three antipsychotics. In patients prescribed 100 mg/day or higher, detection rate of the parent drug was 100% in samples collected both *as presented* and *after washing*. Across all participants, successful detection of CLZ correlated with daily dose, where only one sample was CLZ negative. This sample was collected *after washing* from participant AP-109, prescribed the lowest dose of 25 mg/day. These results indicate that the fingerprint test has sufficient sensitivity for medical application, for example, in the treatment of schizophrenia, where a low dose of clozapine is 150—300 mg/day (Subramanian et al., 2017). Using presence of parent drug in at least one finger *after washing* as indication of drug administration, the finger sweat test was 100% effective in monitoring CLZ adherence in all patients, including those prescribed below the typical dose range.

Presence of NDMC within patient samples was less prevalent and did not correlate with prescribed dose. Although not detected in any samples from AP-109, other patients also did not exhibit the metabolite in any samples *after washing*, namely, AP-127 and AP-118, prescribed 140 and 500 mg/day. It is possible that these results are related to quality of sample, as previous studies in finger sweat have shown that poor deposition of sample can generate false negative results (Ismail et al., 2022). Using presence of the metabolite in at least one finger as indication of adherence in patients prescribed 100 mg/day or higher, the test was 94% effective using samples *as presented* or 88% effective *after washing*.

#### 3.1.2 Quetiapine

Both QTP and NQTP were detected in all samples collected *as presented* and *after washing*. Therefore, the test was 100% effective in monitoring QTP adherence. Although only a small number of patients were recruited, these results suggest the test is sufficiently sensitive to monitor typical doses prescribed in the treatment of bipolar disorder and schizophrenia which range 400—800 mg/day by oral administration (Muneer, 2015) compared to 100—500 mg/day prescribed to patients included in this study.

#### 3.1.3 Olanzapine

OLZ and its metabolite performed most inconsistently of the three groups. Whilst OLZ was detected in 75% of samples *after washing*, DMO was rarely detected and only observed in *as presented* samples. This is likely due to the higher LOD (100 pg) and low doses of drug in comparison to others within this study. Inspection of the data per participant showed that 100% (n = 20) could be classified as ‘*Positive*’ based on detection of OLZ in at least one finger *as presented* or 80% *after washing*. Detection of OLZ was found to be more consistent in patients who were administered medication by monthly injection, with 100% detection of OLZ in all fingers both before and after washing.

### 3.2 Drug contact *versus* administration

To explore the possibility of using the test to monitor adherence to antipsychotic medication, we assessed the feasibility of using the test to distinguish between contact and ingestion of a given drug. It is possible that false positive results could arise from both innocent handling of a tablet, as well as a deliberate attempt to cheat the test. Therefore, finger sweat samples were collected from volunteers who were asked to handle whole tablets with their right hand to mimic administration and to rub the powder crushed tablets across the tips of the left fingers to simulate intentional doctoring of a result.

**FIGURE 2:**
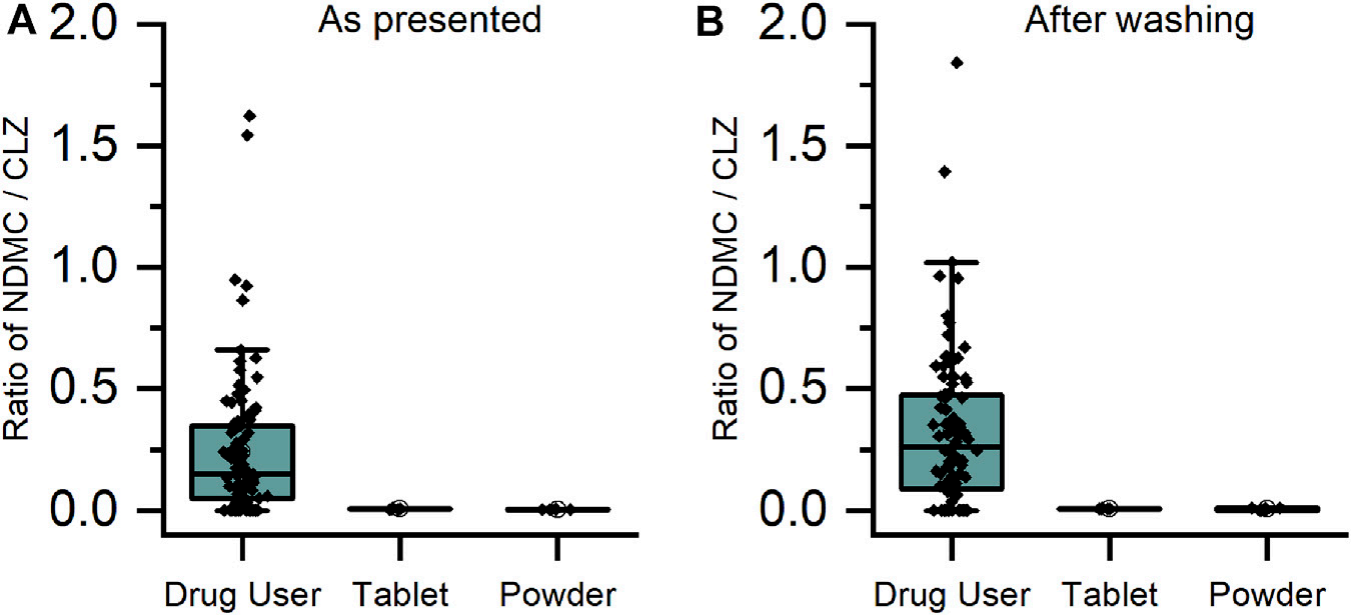
Ratio of metabolite/parent drug in finger sweat samples collected **(A)** “as presented” and **(B)** “after washing” for clozapine users prescribed ≥100 mg/day (n = 32 participants, labelled “drug user”) and volunteers (N = 2) which held antipsychotic tablet in right hand (labelled “tablet”) and rubbed crushed tablet in left hand (labelled “powder”).

**FIGURE 3:**
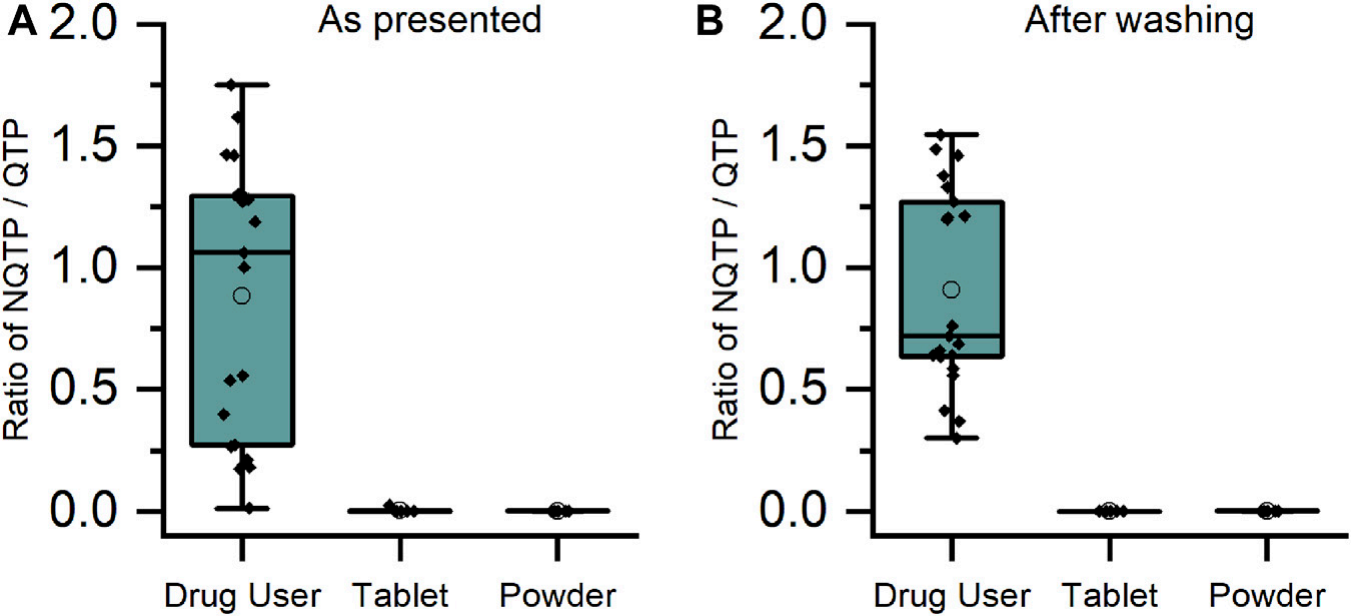
Ratio of metabolite/parent drug in finger sweat samples collected **(A)** “as presented” and **(B)** “after washing” for quetiapine users (n = 7 participants, labelled “drug user”) and volunteers (N = 2) which held antipsychotic tablet in right hand (labelled “tablet”) and rubbed crushed tablet in left hand (labelled “powder”).

For CLZ and QTP, contact-only participants were distinguished from drug users by lower levels or lack of metabolite within their sweat samples. Through plotting the ratio of metabolite to parent drug, a visually and statistically significant (*p* < 0.001, 2-tailed Mann Whitney *U*-test) distinction between the two populations was observed both before and after hand washing (Figures 2, 3).

As DMO was rarely detected in either the drug user or the contact only samples, it was not possible to adopt the same approach as for CLZ and QTP. Comparison of the mass of parent drug showed a statistically significant (*p* < 0.001, 2-tailed Mann Whitney *U*-test) difference between the two populations for OLZ (see Supplementary Figure S4). This result was repeatable for CLZ and QTP also, suggesting that high levels of parent drug could indicate a false positive result originating from contact with medication.

### 3.3 Comparison of plasma and finger sweat

Paired plasma and finger sweat samples were collected from a subgroup of patients prescribed CLZ. Daily dose was matched to the previous analysis, ranging 25—550 mg/day. Given that many of the first batch of samples exhibited analyte levels above the calibration range, the linear range was extended to provide quantitative measurements of drug and metabolite in finger sweat.

Finger sweat was found to be 100% effective in monitoring adherence, where both CLZ and NDMC were detected in at least one finger either *as presented* and *after washing*. Only one participant (PS-005) provided any negative finger sweat samples. In this case, the parent drug was detected in two fingers *as presented* and a single finger *after washing*, whereas NDMC was detected in the index finger *as presented* only. This could be due to several factors: including the sub-therapeutic dose of CLZ (25 mg/day), the short duration of treatment (approximately 1 week) and poor deposition of sample. It should also be noted that this patient was difficult to bleed, suggestive of dehydration, resulting in insufficient whole blood for plasma separation. In such cases, where an indication of patient adherence is required but collection of blood has failed, finger sweat provides a simplistic opportunity to assess adherence in the absence of sufficient plasma for drug analysis.

**FIGURE 4:**
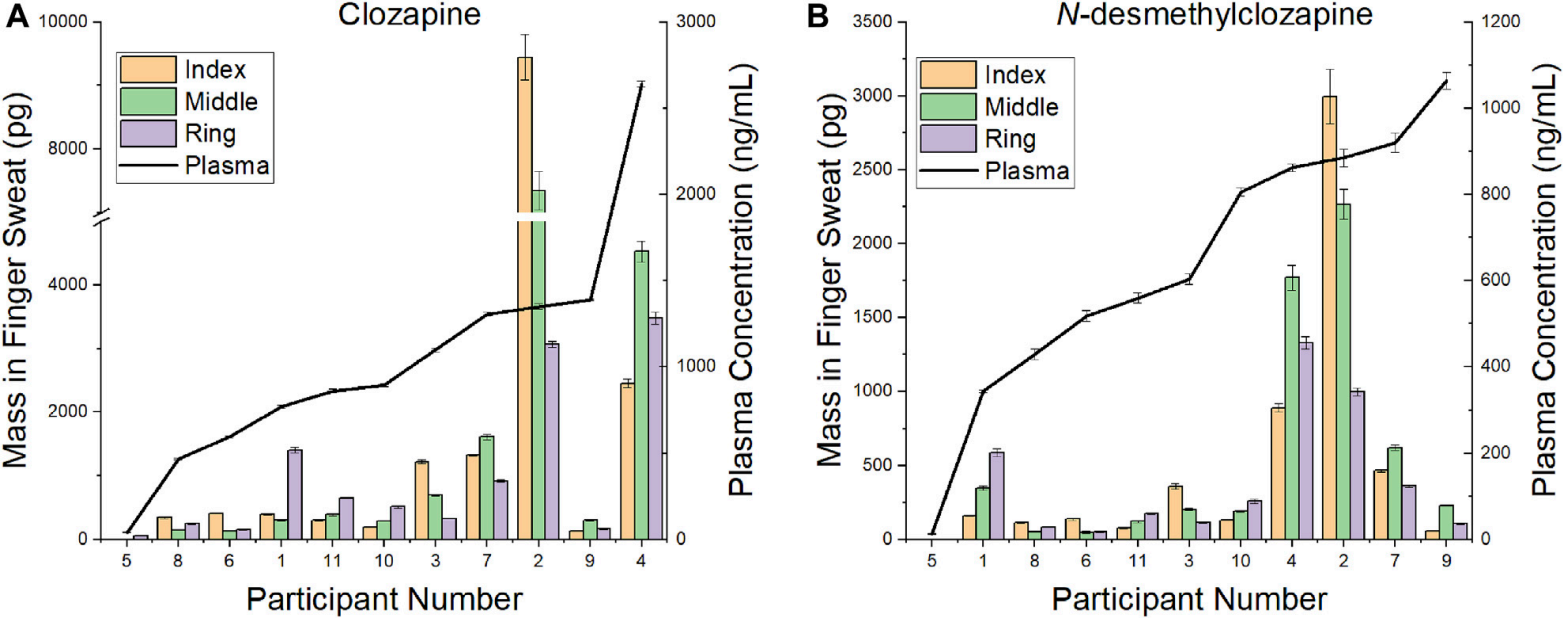
Comparison of mass in finger sweat collected “after washing” *versus* plasma concentration for **(A)** clozapine and **(B)** *N*-desmethylclozapine.

**FIGURE 5:**
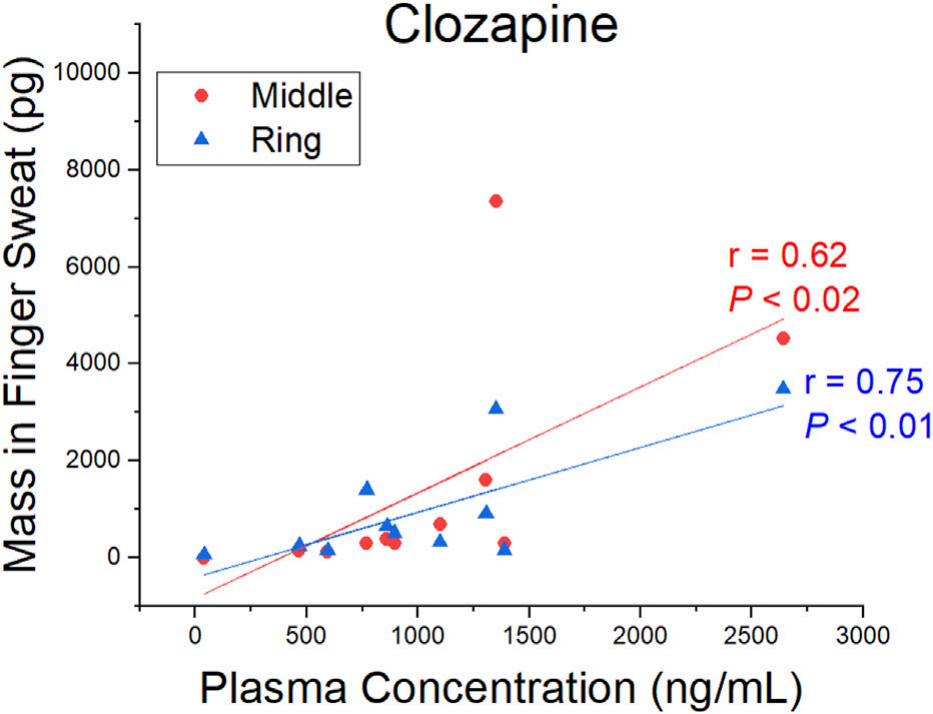
Pearson correlation coefficient (r) and *p*-value (*P*) (1-tailed) for the mass of clozapine in finger sweat collected “after washing” from the middle and ring fingers and clozapine concentration in paired plasma samples.

A comparison of CLZ and NDMC in *after washing* samples *versus* concentration in plasma is displayed in Figure 4. The ratio of metabolite to parent drug in sweat was 0.42 ± 0.19 compared to 0.65 ± 0.22 in plasma. Visually, the mass of analyte in finger sweat was found to mirror the concentration in plasma. The correlation of average mass per finger *versus* plasma concentration yields a Pearson correlation coefficient (r) of 0.56 (*p* < 0.04, 1-tailed) and 0.45 (*p* < 0.09, 1-tailed) for CLZ and NDMC. Inspection of CLZ correlation per finger showed that the middle and ring finger correlated most strongly, with Pearson’s r values of 0.62 (*p* < 0.02, 1-tailed) and 0.75 (*p* < 0.01, 1-tailed) (Figure 5). This suggests that sampling of middle and ring fingers is preferable for best representation of plasma level.

## 4 Discussion

Antipsychotic medication is considered to be the main course of treatment of psychotic disorders. Despite the extreme consequences associated with nonadherence and consequent relapse, patient adherence is often assessed by subjective means (Velligan et al., 2006). The introduction of a simple and dignified method of monitoring patients could improve patient experience and outcomes. Collection of finger sweat is quick, convenient and does not require any specialist training or equipment. Targeted analysis of finger sweat samples offers a more accurate alternative to subjective adherence monitoring methods without the invasive procedures required for blood collection. With further development, finger sweat based diagnostics has the potential to inform clinicians on dose efficacy and toxicity.

In this work, three common antipsychotic drugs (CLZ, QTP, OLZ) and their metabolites were successfully detected in finger sweat using LC-MS/MS. Our results demonstrate that a test based on the detection of the parent drug in at least 1 of 3 samples collected *as presented* was 100% effective in monitoring patient adherence. Using participants who had handled antipsychotic medication only, we were able to distinguish between contact and administration of a given drug where potential false positive results were characterized either by a lack of metabolite, or by exceedingly high levels of parent drug. Similarly, no interferences were found in the negative control group. A limitation of the finger sweat sampling method used within this study was additional time required for handwashing and collection of *after washing* samples. These results suggest that for qualitative assessment of adherence using finger sweat, the addition of the hand washing step is not necessary. This would simplify the test for use by a layperson, as well as further speed the collection process.

To our knowledge, this is also the first study describing the relationship of CLZ and its metabolite in finger sweat and plasma. Although inter-finger levels varied, the test was sufficiently sensitive to detect the typical dosing range of CLZ prescribed in the treatment of psychotic disorders such as schizophrenia. The middle and ring fingers were found to reflect the plasma concentrations most strongly, where a statistically significant correlation between CLZ plasma concentration and mass per finger *after washing* was observed. These data provide a foundation for further exploration of the relationship between the two biological matrices. Opportunities include the implementation of a standardization procedure, such as that described by (Goucher et al., 2009) whereby creatinine was used to smooth the elimination profile of lorazepam in ten overlayed fingerprints from the same donor or by (Ismail et al., 2022) who employed taurine to reduce the coefficient of variation of acetyl isoniazid (the main metabolite of a key anti-tuberculosis drug) between multiple fingerprint samples collected simultaneously from the same donor. Identification and implementation of similar small molecule metabolites within the antipsychotic finger sweat workflow could account for intra-donor variability and allow for more reliable quantitative measurements in future analyses.

In conclusion, a sensitive, convenient, and non-invasive method for monitoring adherence in antipsychotic patients has been developed. Analysis of human finger sweat samples from drug users has shown the method to be 100% effective at detecting commonly prescribed doses of antipsychotic medication, given the test criteria (detection of parent drug in 1 of 3 samples). Using these criteria, the test can distinguish drug administration from drug contact, and non-drug users. The finger sweat technology has broader opportunities in clinical environment, in quantitative monitoring antipsychotic medication or monitoring adherence to other treatment regimes.

## Data Availability

The raw data supporting the conclusion of this article will be made available by the authors, without undue reservation.
