# Supplementary material for: Noninvasive drug adherence monitoring of antipsychotic patients via finger sweat testing
Source: Front Chem. 2023 Aug 31;11:1245089. doi: 10.3389/fchem.2023.1245089 (PMC10500062; doi:10.3389/fchem.2023.1245089)
Supplement: Supplementary file 1 [file DataSheet1.PDF]

Supplementary Material

Noninvasive drug adherence monitoring of antipsychotic patients via finger sweat testing

K. Longman, C. Frampas, H. Lewis, C. Costa, R. Nilforooshan, M. Chambers, and M. Bailey*

*** Correspondence:** Professor Melanie Bailey: m.bailey@surrey.ac.uk

1 Patient Metadata

Supplementary Table 1. Summary of all participants included within this study, including patients, contact only and negative control group.

Population	Participant ID	Antipsychotic Drug	Dose (mg) [1]	Time since last dose (hrs)	Finger Sweat Type [2]
Patients (finger sweat only)	AP-038	Olanzapine	20	16	AP, AW
	AP-039	Quetiapine	300	16.5	AP, AW
	AP-040	Olanzapine	10	17	AP, AW
	AP-041	Olanzapine	20	18	AP, AW
	AP-042	Quetiapine	300	4	AP, AW
	AP-044	Olanzapine	20	20	AP, AW
	AP-045	Clozapine	375	19	AP, AW
	AP-046	Quetiapine	100	6	AP, AW
	AP-047	Olanzapine	7.5	19	AP, AW
	AP-048	Quetiapine	300	20	AP, AW
	AP-050	Quetiapine	Unknown	Unknown	AP, AW
	AP-104	Clozapine	250	21	AP, AW
	AP-105	Clozapine	250	19	AP, AW
	AP-109	Clozapine	25	6.5	AP, AW
	AP-110	Olanzapine	300 *	66	AP, AW
	AP-111	Clozapine	250	18.5	AP, AW
	AP-112	Clozapine	100	7	AP, AW
	AP-113	Clozapine	150	7	AP, AW
	AP-115	Clozapine	250	12	AP, AW
	AP-116	Clozapine	125	12	AP, AW
	AP-117	Clozapine	100	< 1	AP, AW
	AP-118	Clozapine	500	5.5	AP, AW
	AP-119	Clozapine	275	5	AP, AW
	AP-120	Clozapine	200	< 1	AP, AW
	AP-121	Clozapine	200	11.5	AP, AW
	AP-122	Clozapine	275	12	AP, AW

	AP-123	Clozapine	200	15.5	AP, AW
	AP-124	Clozapine	100	6.5	AP, AW
	AP-125	Clozapine	350	18.5	AP, AW
	AP-126	Clozapine	200	2.5	AP, AW
	AP-127	Clozapine	140	14	AP, AW
	AP-128	Olanzapine	20	14.5	AP, AW
	AP-129	Clozapine	350	13	AP, AW
	AP-130	Olanzapine	2.5	13	AP, AW
	AP-131	Olanzapine	10	25.5	AP, AW
	AP-132	Olanzapine	20	16	AP, AW
	AP-133	Olanzapine	20	16.5	AP, AW
	AP-134	Olanzapine	15	13	AP, AW
	AP-135	Olanzapine	15	14.5	AP, AW
	AP-136	Clozapine	100	5	AP, AW
	AP-137	Clozapine	275	14	AP, AW
	AP-139	Olanzapine	420 *	< 1	AP, AW
	AP-140	Olanzapine	300 *	< 1	AP, AW
	AP-141	Quetiapine	500	26	AP, AW
	AP-142	Clozapine	225	5.5	AP, AW
	AP-143	Olanzapine	300 *	< 1	AP, AW
	AP-144	Olanzapine	405 *	< 1	AP, AW
	AP-146	Quetiapine	100	16.5	AP, AW
	AP-147	Olanzapine	300 *	24	AP, AW
	AP-148	Olanzapine	405 *	< 1	AP, AW
	AP-149	Olanzapine	405 *	< 1	AP, AW
	AP-151	Clozapine	250	24	AP, AW
	AP-152	Clozapine	200	13	AP, AW
	AP-153	Clozapine	275	24	AP, AW
	AP-154	Clozapine	300	3	AP, AW
	AP-155	Clozapine	150	19	AP, AW
	AP-156	Clozapine	200	13.5	AP, AW
	AP-157	Clozapine	200	13	AP, AW
	AP-158	Clozapine	300	2	AP, AW
	AP-159	Clozapine	250	12	AP, AW
Plasma subgroup	PS-001	Clozapine	100	16	AP, AW, P
	PS-002	Clozapine	450	16	AP, AW, P
	PS-003	Clozapine	275	14	AP, AW, P
	PS-004	Clozapine	550	14	AP, AW, P
	PS-005	Clozapine	25	11.5	AP, AW, P
	PS-006	Clozapine	500	15.5	AP, AW, P
	PS-007	Clozapine	550	12	AP, AW, P
	PS-008	Clozapine	400	11	AP, AW, P
	PS-009	Clozapine	300	14.5	AP, AW, P
	PS-010	Clozapine	500	1.5	AP, AW, P
	PS-011	Clozapine	300	15	AP, AW, P

Contact only	CO-003	Quetiapine	-	-	AP, AW
	CO-004	Quetiapine	-	-	AP, AW
	CO-005	Olanzapine	-	-	AP, AW
	CO-006	Olanzapine	-	-	AP, AW
	CO-007	Clozapine	-	-	AP, AW
	CO-008	Clozapine	-	-	AP, AW
Negative control group	BG-001 to -030	-	-	-	Right index only: AP, AW

[1] mg per day (orally), excluding monthly depot injection where samples marked as *.
[2] (AP) finger sweat as presented, (AW) finger sweat after hand washing and (P) plasma.

2 Mass Spectrometer Operating Conditions

Supplementary Table 2. Operating conditions of the Thermo Scientific™ Q Exactive™ Plus Hybrid Quadrupole-Orbitrap™ Mass Spectrometer.

**Parameter**	**Operating Condition**
Spray voltage	4.0 kV
Capillary temperature	320 °C
S-lens RF level	50
Sheath gas flow rate	35
Aux gas flow rate	8
Scan range	*m/z* 100 to *m/z* 500
Resolution	70 000 at *m/z* 200
Polarity	Positive
AGC target	1e6
Maximum inject time	200
**MS/MS Parameter**	**Operating condition**
Mode	FSMS / dd-MS^2 with inclusion lists
Resolution	35 000 at *m/z* 200
Loop Count	2
Normalised Collision Energy	30, 70, 110
Dynamic Exclusion	10 seconds

3 Finger Sweat Method Performance Data

3.1 Selectivity

(A)

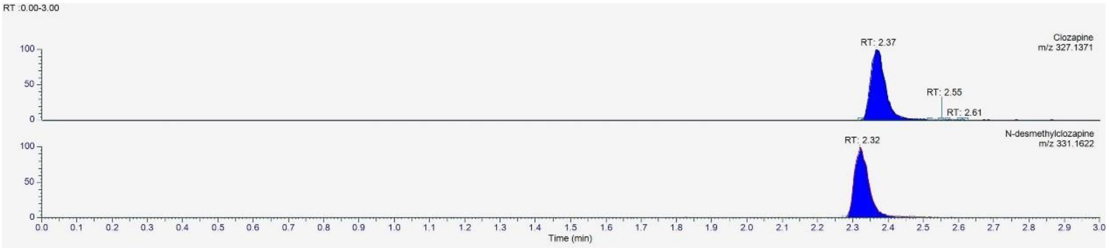

(B)

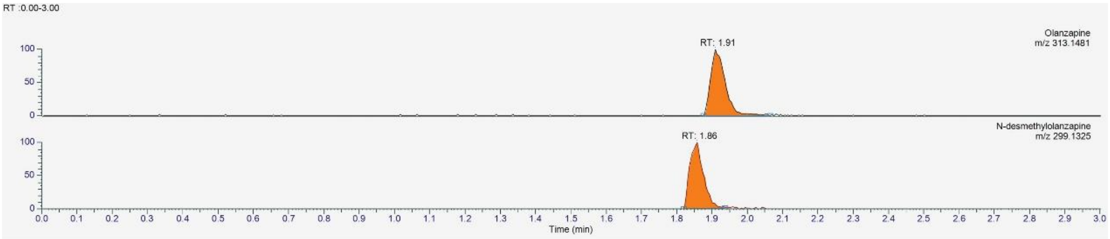

(C)

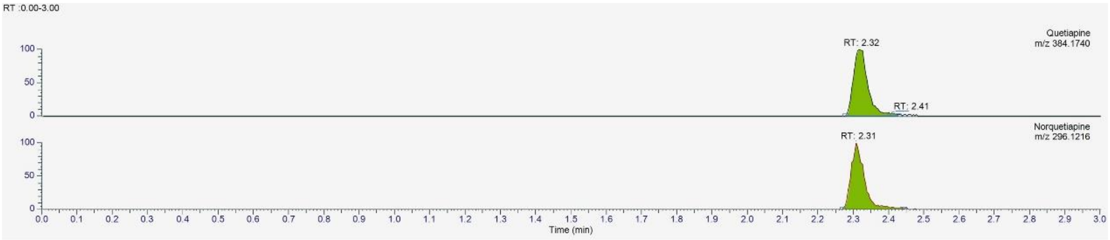

Supplementary Figure 1. Ion extracted chromatograms for (A) clozapine and N-desmethylclozapine, (B) olanzapine and N-desmethylolanzapine and (C) quetiapine and norquetiapine in standard solution.

3.2 Linearity and LOD

Supplementary Table 3. The limit of detection, working range and associated R^2 values for all antipsychotic drugs and metabolites extracted from paper. The LOD is defined as the lowest mass extracted from paper which could be determined with a RSD < 20%.

Analyte	Internal Standard (pg)	LOD (pg)	Working Range (pg)	R^2
Clozapine	200; 350	30	60 – 360; 100 – 600	> 0.990; > 0.992
N-desmethylclozapine	200; 350	50	60 – 360; 100 – 600	> 0.973; > 0.995
Olanzapine	350	50	100 – 600	> 0.993
N-desmethylolanzapine	350	100	100 – 600	> 0.989
Quetiapine	100	10	30 – 180	> 0.986
Norquetiapine	100	20	30 – 180	> 0.994

3.3 Accuracy and precision

Supplementary Table 4. Precision data for finger sweat methodology: qualitative analysis of clozapine, *N*-desmethylclozapine. olanzapine, *N*-desmethylolanzapine, quetiapine and norquetiapine QC samples using LC-MS.

Analyte	Mass deposited on paper (pg)	Intra-day		Inter-day	
		Analyte-to-internal standard ratio [a]	RSD (%)	Analyte-to-internal standard ratio [a]	RSD (%)
CLZ	90	0.509 ± 0.009	1.78	0.513 ± 0.005	0.99
	200	1.176 ± 0.014	1.15	1.185 ± 0.033	2.82
	330	1.923 ± 0.039	2.04	1.885 ± 0.014	0.74
NDMC	90	0.439 ± 0.008	1.79	0.442 ± 0.024	5.35
	200	0.995 ± 0.002	0.17	1.009 ± 0.032	3.13
	330	1.603 ± 0.033	2.07	1.598 ± 0.116	7.28
OLZ	150	0.678 ± 0.019	2.79	0.668 ± 0.043	6.40
	350	3.459 ± 0.051	1.49	3.263 ± 0.974	29.83
	550	6.694 ±0.173	2.58	6.077 ± 1.622	26.69
DMO	150	0.193 ± 0.015	7.81	0.237 ± 0.083	34.96
	350	1.522 ± 0.050	3.30	1.571 ± 0.389	24.75
	550	3.218 ± 0.067	2.08	3.037 ± 0.616	20.29
QTP	40	0.199 ± 0.001	0.30	0.203 ± 0.004	1.99
	100	0.540 ± 0.008	1.51	0.550 ± 0.011	2.02
	160	0.843 ± 0.018	2.18	0.828 ± 0.006	0.71
NQTP	40	0.261 ± 0.002	0.69	0.260 ± 0.015	5.58
	100	0.698 ± 0.006	0.91	0.737 ± 0.057	7.80
	160	1.084 ± 0.022	2.02	1.124 ± 0.141	12.50

[a] Results are expressed as mean ± standard deviation (n = 9 papers).

Supplementary Table 5. Accuracy and precision data for finger sweat methodology at the higher working range: analysis of clozapine and *N*-desmethylclozapine QC samples using LC-MS.

Analyte	Mass deposited on paper	Intra-day			Inter-day		
		Calculated mass [a]	RSD	RE	Calculated mass [a]	RSD	RE
CLZ	150	157.7 ± 0.6	0.4	5.2	157.3 ± 4.5	2.9	4.9
	350	340.3 ± 2.8	0.8	-2.8	345.5 ± 6.6	1.9	-1.3
	550	522.0 ± 17.2	3.3	-5.1	534.0 ± 13.8	2.6	-2.9
NDMC	150	159.9 ± 0.6	0.4	6.6	158.2 ± 3.3	2.1	5.5
	350	346.9 ± 2.2	0.6	-0.9	351.0 ± 7.3	2.1	0.3
	550	525.9 ± 16.6	3.2	-4.4	534.8 ± 20.5	3.8	-2.8

[a] Results are expressed as mean ± standard deviation (n = 9 papers). All masses are stated as pg, RSD and RE are expressed as %.

3.4 Recovery and matrix effect

Recovery from paper was assessed using a mid-range calibrator for each analyte. Recovery was >70% for CLZ/NDMC, >94% for OLZ/DMO at 300 pg, and >64% for QTP/NQTP at 90 pg. To evaluate matrix effect, analyte standard samples (500 pg) were extracted in the presence of finger sweat *as presented* and *after washing* from three donors. Matrix effect was <10% for antipsychotics and metabolites.

3.5 Carryover

Using injections on blank mobile phase after the highest calibrator standard, carryover was not observed.

3.6 Stability

Stability samples (100 pg on paper) were extracted and analysed over 48 hours whilst stored in the autosampler (5 °C). Stability of extracted samples was found to be sufficient for batch analysis (analyte-to-internal standard RSD < 15% for all analytes).

4 Plasma Method Performance Data

4.1 Selectivity

No interferences were observed within blank pooled plasma samples.

4.2 Linearity

Mean linearity of R^2 > 0.9991 and > 0.9985 for clozapine and N-desmethylclozapine across three validation runs.

4.3 LOD and LLOQ

Clozapine and its metabolite detected as low as 10 ng/mL in spiked plasma; however, using validation guidelines, LLOQ was 25 ng/mL (RSD and RE < 20%).

4.4 Accuracy and precision

Supplementary Table 6. Accuracy and precision data for plasma methodology: analysis of clozapine and *N*-desmethylclozapine QC samples using LC-MS.

**Analyte**	**Added concentration**	**Intra-day**			**Inter-day**		
		**Calculated concentration**	**RSD**	**RE**	**Calculated concentration**	**RSD**	**RE**
CLZ	75	70.9 ± 0.2	1.7	-5.5	72.6 ± 1.7	1.9	-3.2
	500	479.4 ± 10.6	1.0	-4.1	494.6 ± 4.3	0.7	-1.09
	1200	1243.6 ± 24.5	1.0	3.6	1236.3 ± 34.9	0.5	3.0
NDMC	75	64.4 ± 0.3	5.3	-14.1	65.8 ± 2.3	3.6	-12.3
	500	476.6 ± 24.1	3.6	-4.7	513.7 ± 11.6	2.2	2.7
	1200	1249.9 ± 30.1	0.9	4.2	1255.3 ± 55.1	2.0	4.6

[a] Results are expressed as mean ± standard deviation (n = 3). All concentrations are stated as ng/mL, RSD and RE are expressed as %.

4.5 Carryover

Carryover was only observed over 500 ng/mL where it was < 1% and therefore deemed negligible.

5 MS/MS Data

(A)

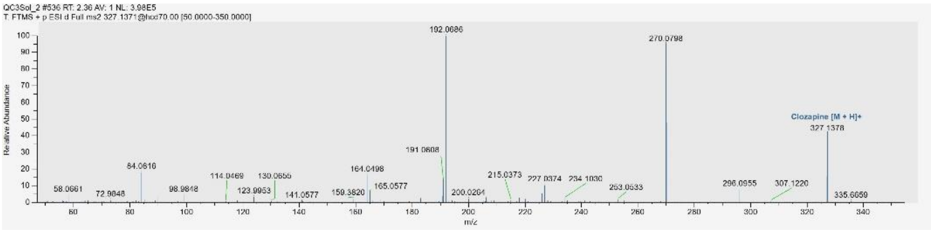

(B)

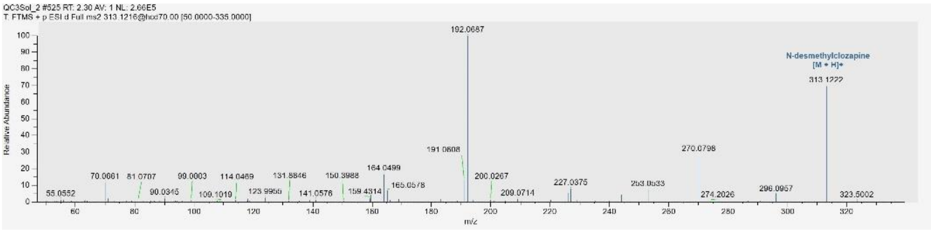

(C)

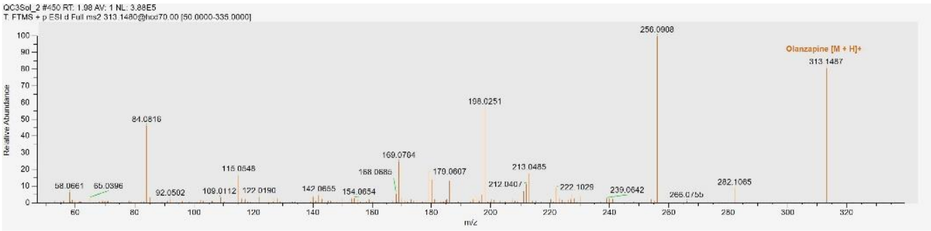

(D)

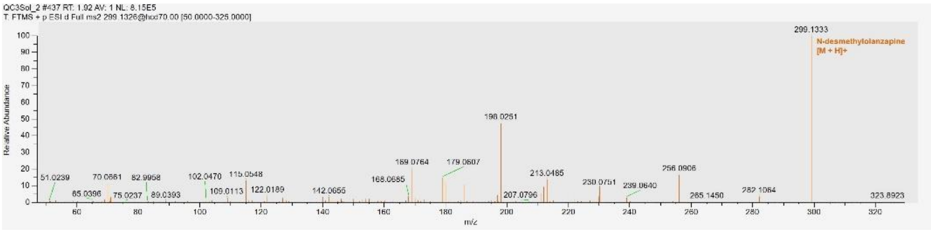

(E)

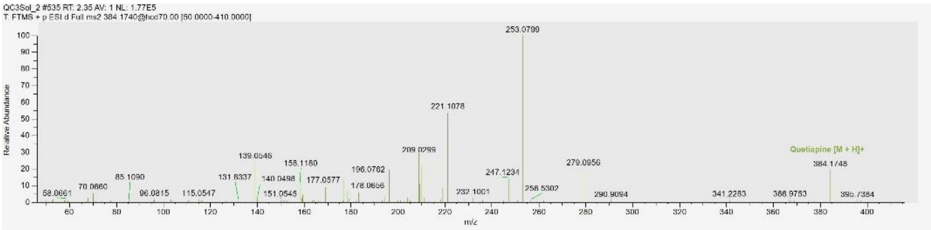

(F)

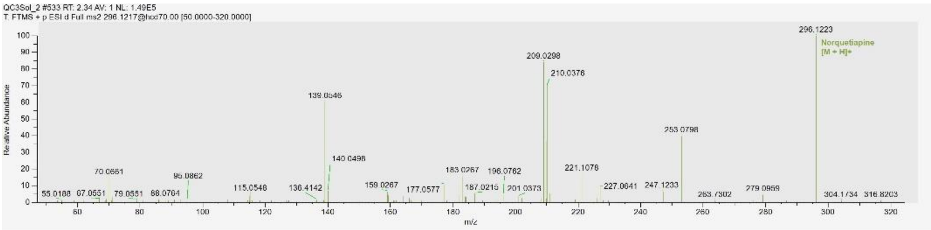

Supplementary Figure 2. Example MS/MS spectra for (A) clozapine, (B) N-desmethylclozapine, (C) olanzapine, (D) N-desmethylolanzapine, (E) quetiapine and (F) norquetiapine from standard solution.

6 Negative Control Group Data

(A) Clozapine:

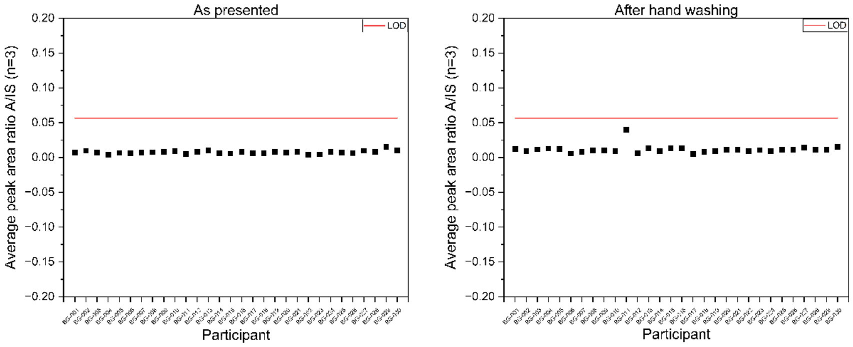

(B) N-desmethylclozapine:

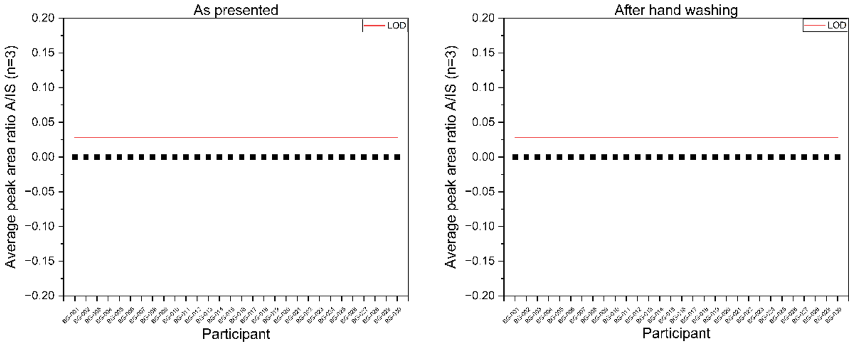

(C) Olanzapine:

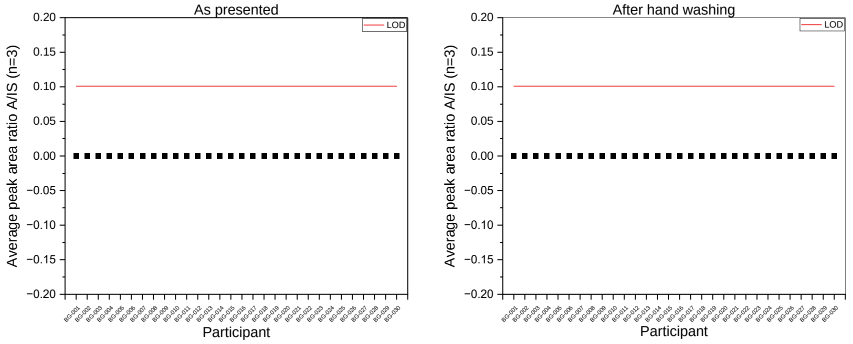

(D) N-desmethylolanzapine:

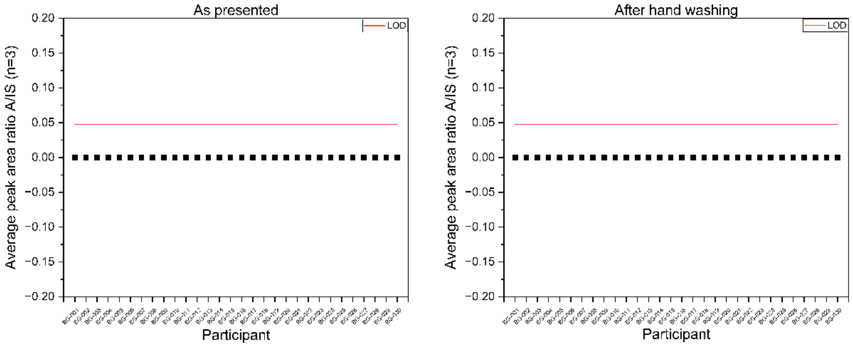

(E) Quetiapine:

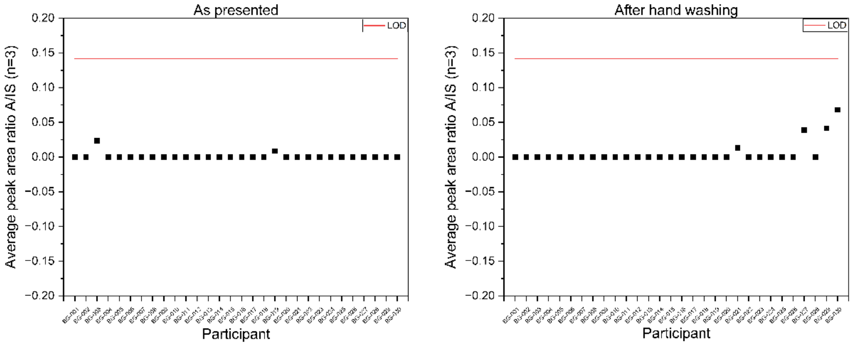

(F) Norquetiapine:

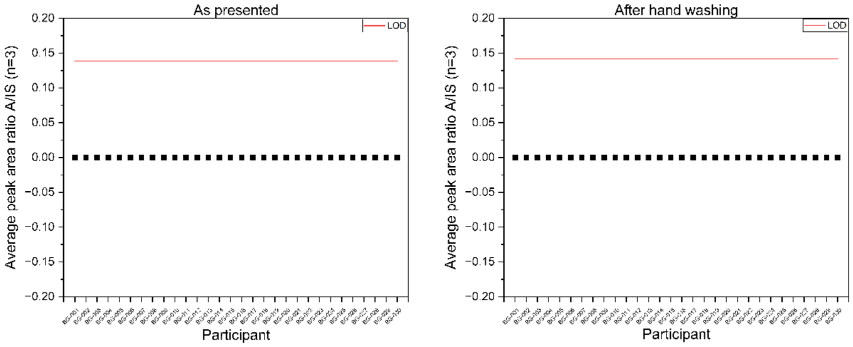

Supplementary Figure 3. Finger sweat screening results for (A) clozapine, (B) N-desmethylclozapine, (C) olanzapine, (D) N-desmethylolanzapine, (E) quetiapine and (F) norquetiqpine from the negative control group for samples collected “as presented” and “after washing” (n = 1 sample per participant per condition).

7 Drug Contact Versus Administration Data

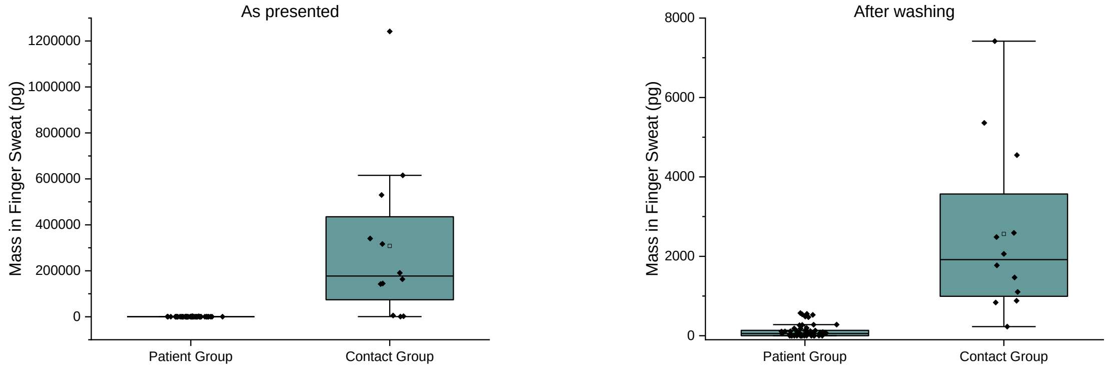

Supplemental Figure 4. Mass of olanzapine in finger sweat in samples collected 'as presented' and 'after washing' from n = 20 patients and N = 2 volunteers. Contact study includes samples from both left and right hands, used to handle the tablet (right hand) and to rub the powder into the fingertips (left hand).
